# A case report of Wilson's disease with rare frontal lobe white matter lesions

**DOI:** 10.3389/fped.2026.1874872

**Published:** 2026-10-29

**Authors:** Xianghui Du, Hu Guo, Le Ding, Chao Liang, Gang Zhang, Nooraynee Bibi Needah Ginowree

**Affiliations:** Department of Neurology, Children's Hospital of Nanjing Medical University, Nanjing, China

**Keywords:** ATP7B gene, leukoencephalopathy, magnetic resonance imaging, pediatric neurology, Wilson's disease

## Abstract

Wilson's disease (WD) is an autosomal recessive disorder of copper metabolism, typically presenting with hepatic dysfunction and bilateral basal ganglia abnormalities on magnetic resonance imaging (MRI). We report an unusual pediatric case of WD in a 13-year-old boy who presented with subtle neuroregression over the course of one year and recent focal seizures. Magnetic resonance imaging revealed prominent frontal-dominant leukoencephalopathy, which initially raised suspicion for Alexander disease. Brain MRI demonstrated extensive, symmetric T2-weighted and FLAIR hyperintensities predominantly affecting the frontal white matter, with involvement extending to the basal ganglia, thalamus, and brainstem. Notable imaging findings included cystic changes within the frontal lesions and areas of restricted diffusion in the cortical and subcortical regions on diffusion-weighted imaging (DWI). The diagnosis was confirmed by the presence of Kayser-Fleischer rings, low serum ceruloplasmin levels, elevated 24-hour urinary copper excretion, and identification of a homozygous pathogenic *ATP7B* mutation (c.2333G > T). This case demonstrates that WD can radiologically mimic leukodystrophies, underscoring the importance of considering WD in the differential diagnosis of pediatric leukoencephalopathy—even in the absence of classic hepatic or pronounced extrapyramidal findings.

## Introduction

Wilson's disease (WD) is an autosomal recessive disorder of copper metabolism caused by biallelic pathogenic variants in the ATP7B gene. The ATP7B protein, a copper-transporting *P*-type ATPase predominantly localized to hepatocyte trans-Golgi networks, mediates copper incorporation into ceruloplasmin and biliary copper excretion. Abnormal or lost *ATP7B* function disrupts these processes, leading to toxic hepatic copper accumulation, oxidative stress, and progressive multiorgan injury (1–3). WD affects all races and ethnicities without sex predominance, typically presenting from adolescence to early adulthood (4).WD mainly affects young individuals (mean age 19.9 years), with hepatic involvement in 72% and acute liver failure in 31%. Overall mortality was 14%. Mental health complications were frequent, with depression in 47.7% and anxiety in 40%. At the national level, most countries demonstrated moderate prevalence (1–4.99 per 100,000). However, in the non-national-wide widespread prevalence levels, several high-prevalence clusters were identified. The prevalence rates within the range of (5–9.99 per 100,000) (5).

Classic neurological WD typically presents with movement disorders and psychiatric symptoms, supported by neuroimaging findings of symmetric T2 hyperintensity involving the basal ganglia, thalamus, and brainstem (6, 7). However, leukoencephalopathy—particularly with a frontal-dominant pattern—is a rare radiological manifestation that may lead to misdiagnosis as a primary leukodystrophy, such as Alexander disease. This case report highlights this diagnostic challenge and underscores the distinctive neuroimaging features that can prompt consideration of WD.

## Case report

### Clinical presentation

A 13-year-6-month-old boy with an unremarkable family history presented with a one-year history of insidious speech deterioration, drooling, academic decline, and social withdrawal. His symptoms were initially attributed to behavioral issues; however, medical attention was sought after he developed intermittent perioral twitching. Neurological examination revealed dysarthria, bradykinesia, mild lower limb spasticity, hyperreflexia, and a short-step gait. Laboratory tests, including liver transaminases, were within normal limits. Notably, retrospective history revealed an episode of acute hepatitis with marked transaminase elevation (ALT >1,000 U/L) at age 5–6 years, which resolved completely.

Urinalysis revealed occult blood (++), proteinuria (+), red blood cell count of 111.7/*μ*L, and mixed red blood cell morphology. Urinary tract ultrasound indicated bilateral renal parenchymal enhancement. Video electroencephalography (VEEG) revealed bilateral frontal pole, frontal, and midline low-to-moderate amplitude spike waves, spike-and-wave complexes, and rhythmic discharges, predominantly on the left side. During the lucid period, two focal electrical seizures arising from the left frontal region were observed. Along with intermittent tremors at the corner of the mouth in the child, these findings raised suspicion of focal epileptic seizures. Oral antiepileptic drugs were recommended to the parents; however, they refused.

### Imaging findings

Non-contrast computed tomography of the head revealed bilateral hypodense areas in the frontal lobes (Figure 1). Subsequent brain magnetic resonance imaging performed on a 3.0 T scanner demonstrated multifocal, bilateral, and symmetrical abnormalities. On T2-weighted and fluid-attenuated inversion recovery sequences, extensive hyperintense signal was observed in the periventricular and deep frontal white matter, corpus callosum, basal ganglia, thalamus, midbrain, and pons (Figures 2A,B). Within the frontal white matter, several foci exhibited central signal loss on FLAIR, indicating cystic encephalomalacia (Figure 2D, arrow). Diffusion-weighted imaging showed patchy regions of restricted diffusion (hyperintense signal) in the frontal cortical and subcortical areas, as well as in the basal ganglia (Figure 2C). Volumetric analysis disclosed mild atrophy of the basal ganglia. Given the pattern of frontal-predominant leukoencephalopathy with brainstem involvement, Alexander disease was considered the primary radiological differential diagnosis.

**Figure 1:**
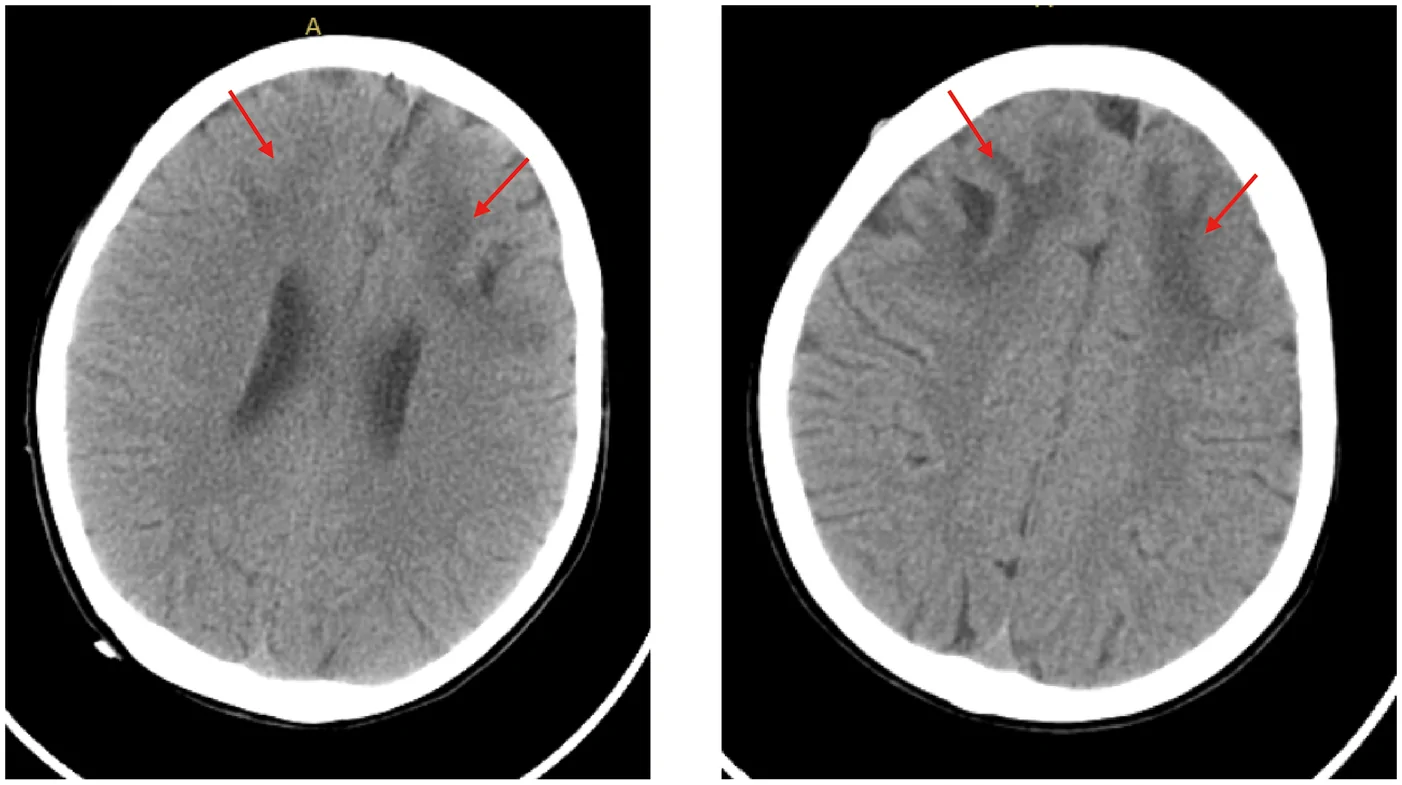
Non-contrast head CT showed bilateral frontal hypodense areas.

**Figure 2:**
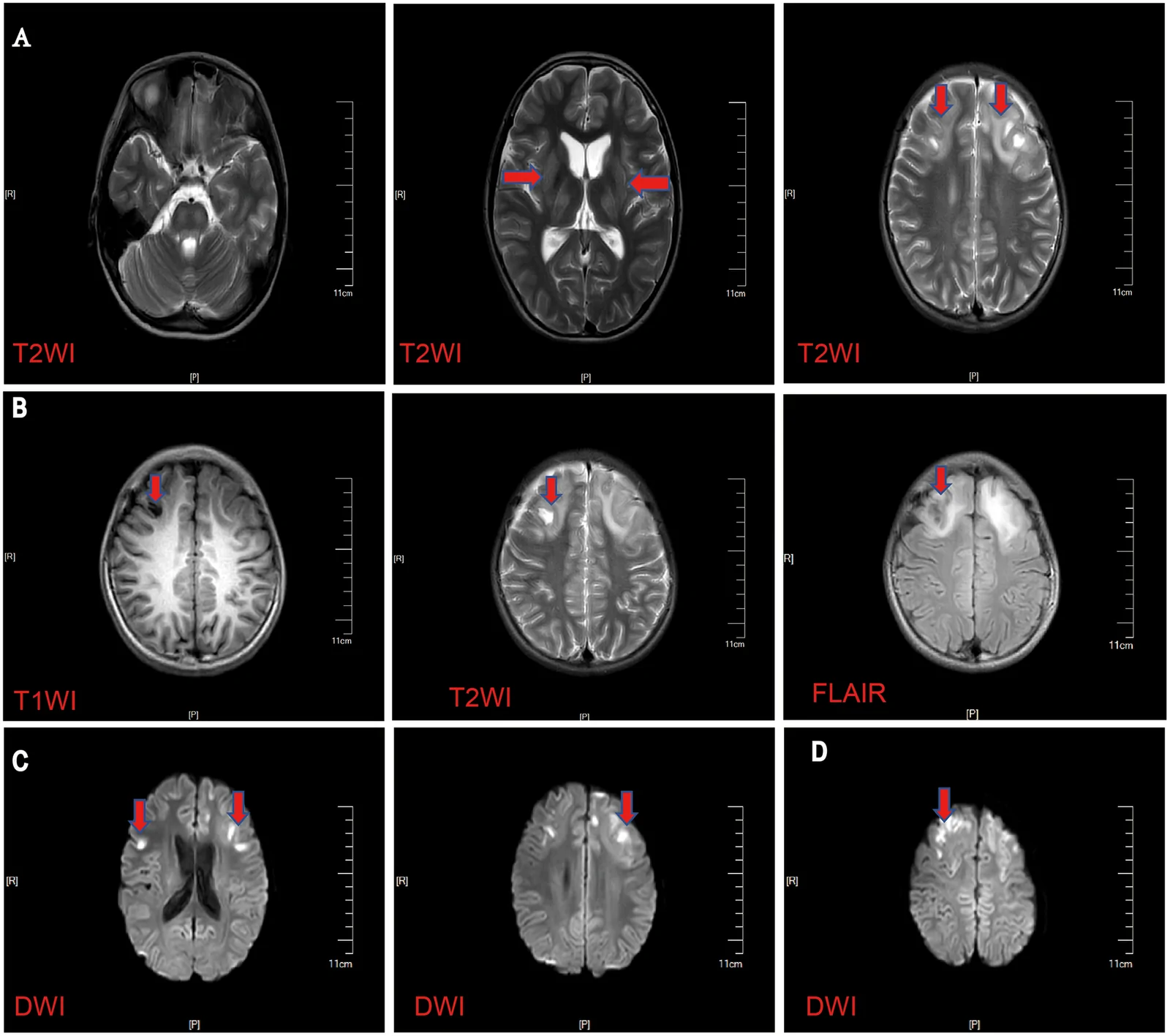
**(A,B)** T1-weighted, T2-weighted and FLAIR images demonstrated extensive hyperintense signal involving the periventricular and deep frontal white matter, corpus callosum, basal ganglia, thalamus, midbrain, and pons. **(C)** Diffusion-Weighted Imaging (DWI) showed patchy areas of restricted diffusion (high signal) in the frontal cortical and subcortical regions, as well as in the basal ganglia. **(D)** Several foci within the frontal white matter showed central signal loss on DWI, consistent with cystic encephalomalacia (arrow).

### Diagnostic work-up and outcome

Whole-exome sequencing identified a homozygous pathogenic variant in *ATP7B* (c.2333G > T, p.Arg778Leu) (Figure 3). The study was approved by the Medical Ethics Committee of our hospital (Ethical Approval Number: 202601013-1). Given the atypical MRI presentation, a broad diagnostic work-up was initiated. Serum ceruloplasmin was severely low (0.0254 g/L; reference range: 0.2–0.6 g/L), and 24-hour urinary copper excretion was elevated (122.0 μg; reference range: 15–60 μg). Slit-lamp examination confirmed the presence of Kayser-Fleischer rings. Using the Leipzig scoring system (8), the patient scored 10 points, confirming the diagnosis of WD. Treatment with a low-copper diet, penicillamine, and zinc sulfate was initiated.

**Figure 3:**
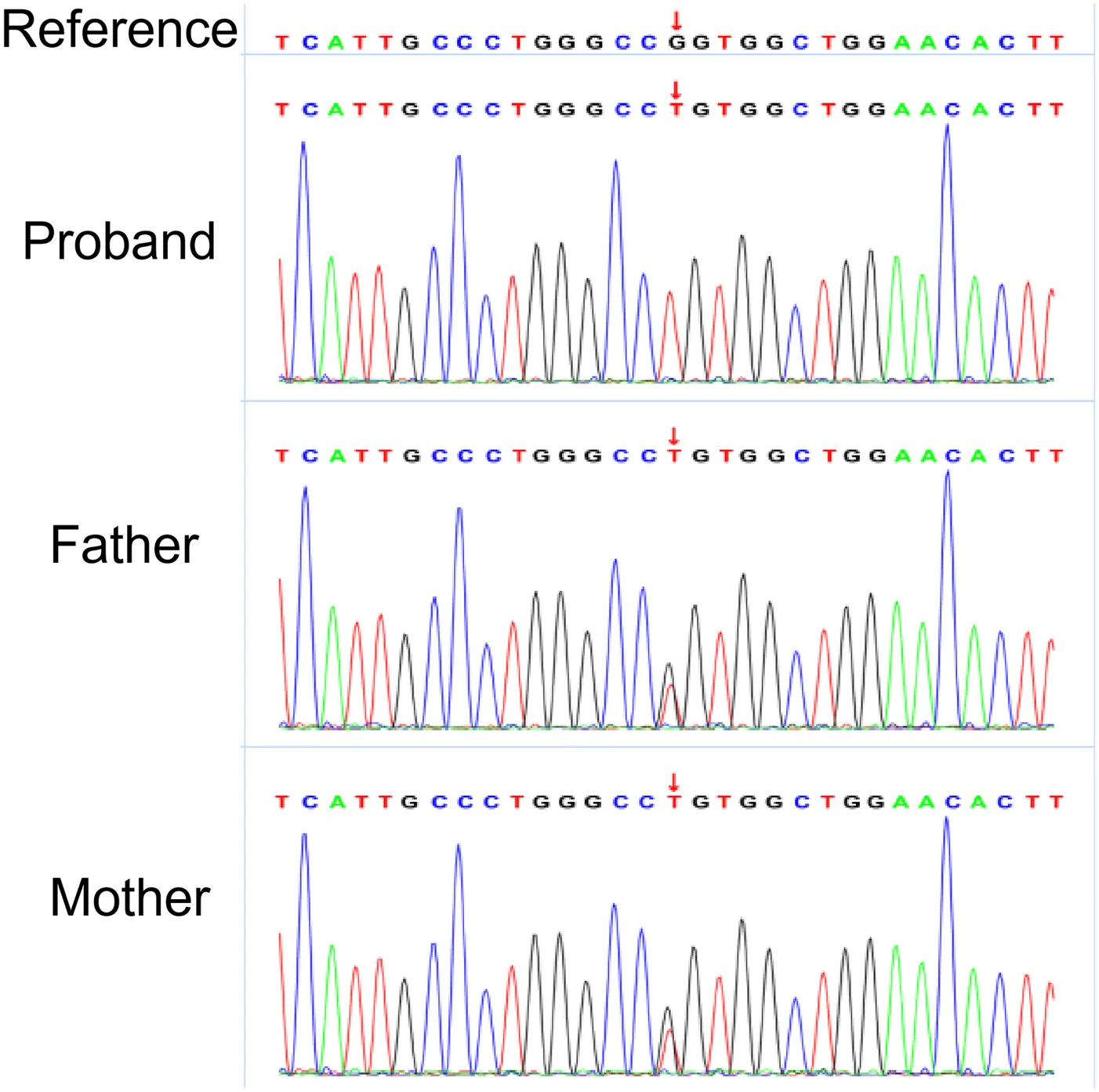
Whole-exome sequencing identified a homozygous pathogenic variant in *ATP7B* (c.2333G > T, p.Arg778Leu). Both the father and mother carry heterozygous c.2333G > T mutation.

Subsequently, the patient was transferred to outpatient follow-up care, primarily due to geographic considerations: the patient's residence was located at a considerable distance from the treatment center. Of note, the patient's parents initially declined antiepileptic pharmacotherapy; results from subsequent telephone follow-up indicated that the parents reported the patient's “clinical symptoms had improved” and did not attend the hospital for further evaluation.

## Discussion

Patients with WD exhibit diverse clinical manifestations that vary depending on the affected organs and severity, primarily involving the liver and/or nervous system (9–11) Neurological presentations predominantly manifest as extrapyramidal dysfunction. Early symptoms are often mild and progress slowly; they may gradually remit or exacerbate in stages, or alternatively, deteriorate rapidly over a few months, leading to severe disability—particularly in younger patients (2, 7). Common neurological manifestations include focal dystonia (e.g., of the vocal cords, speech muscles, or swallowing muscles), which can cause dysphonia, dysarthria, or dysphagia with sialorrhea. Dysarthria is the most frequent neurological symptom, occurring in approximately 85%–97% of cases. Other manifestations include tremors, limb rigidity, bradykinesia, psychiatric and behavioral abnormalities, as well as other rare neurological symptoms (7, 9, 11). A minority of patients may present with choreiform movements, athetosis, or ataxia. WD patients may also exhibit relatively rare epilepsy (12, 13).

In this case, the patient initially presented with drooling and slurred speech over one year prior, raising suspicion of focal dystonia, accompanied by psychomotor abnormalities such as bradykinesia and impaired attention. The parents' insufficient awareness, combined with the mild and insidious symptom onset, led to a lack of treatment until focal epileptic seizures with jaw tremors emerged, prompting eventual medical evaluation.

Usually when a child presents with extensive white matter lesions and softening predominantly affecting the frontal lobe like in this case, the primary clinical consideration should be a leukodystrophy—specifically, Alexander disease (AxD). Other differential diagnoses include metachromatic leukodystrophy and adrenoleukodystrophy. Literature reports suggest that WD may occasionally manifest as frontal lobe-dominant leukoencephalopathy (14, 15); however, such MRI findings are relatively rare in clinical practice. Abnormal signals in the corpus callosum may indicate more extensive brain damage, severe neurological dysfunction, and psychiatric symptoms. In this case, MRI revealed symmetrical abnormal signals in the basal ganglia, thalamus, and brainstem, with significant atrophy observed in the basal ganglia region. Concurrently, softening cystic changes were noted beneath the frontal lobe white matter, suggesting that the coexistence of basal ganglia atrophy and white matter lesions may serve as a critical diagnostic clue for WD. White matter encephalopathy accompanied by cystic changes is even rarer, and cystic changes in WD indicate disease severity or chronic progression, as exemplified in this case. Additionally, cortical hyperintensity on diffusion-weighted imaging (DWI) is relatively uncommon in WD. Sener et al. hypothesized that DWI hyperintensity in the basal ganglia of WD patients results from copper overload-induced cellular damage leading to inflammation, subsequent cellular swelling, and ultimately cell death (15). The multiple cortical hyperintensities observed in this case on DWI may be attributed to cytotoxic edema or copper-associated inflammation.

WD predominantly involving the liver typically manifests at an earlier age (onset may occur before 2 years of age), while neurological involvement often appears 10 years later than hepatic disease (usually after 15 years of age) (11). Patients usually present with varying degrees of liver injury at diagnosis, with mild cases exhibiting only histological damage. Clinical presentations vary depending on severity and disease duration, including asymptomatic cases, acute hepatitis, acute liver failure (ALF), chronic hepatitis, and cirrhosis. Studies indicate that 3%–40% of patients may remain completely asymptomatic (16).

The uniqueness of this case lies in the following: after genetic testing results were obtained, the parents reported a history of acute hepatitis of unknown origin during the patient's preschool years, with peak alanine aminotransferase levels exceeding 1,000 U/L. Neurological symptoms did not emerge until several years later, during which time liver function remained entirely normal, and only mild occult hematuria with renal involvement was observed. The subsequent normalization of transaminase levels may be attributed to relatively mild liver injury in the very early stages of WD or to low levels of copper deposition. Young children possess some capacity for hepatocyte self-repair, which can lead to transient reductions in transaminases. Concurrent viral infections may also contribute to a temporary decline in transaminases following successful control and resolution of the infection, even though the underlying WD pathology persists.

This case serves as a powerful reminder to clinicians: proactive identification of the etiology of hepatitis during the preschool years, early measurement of serum ceruloplasmin and urinary copper, inclusion of WD in the differential diagnosis, and timely initiation of copper chelation therapy could have entirely prevented irreversible neurological dysfunction. Therefore, routine diagnostic evaluation for WD, including differential diagnosis, should be performed in patients presenting with acute hepatitis of unknown origin, liver failure, or isolated extrapyramidal motor disturbances.

WD is a fully treatable neurogenetic disorder, making early detection and accurate diagnosis essential. The therapeutic approach requires lifelong, uninterrupted, individualized therapy aimed at achieving a negative copper balance (6, 17). Management of neurological symptoms involves not only fundamental copper-chelating or zinc therapy but also targeted symptomatic medications—such as trihexyphenidyl, clonazepam, and botulinum toxin—for specific manifestations like tremors and dystonia, along with active rehabilitation interventions (physical therapy, occupational therapy, and speech/swallowing training) (18, 19). If diagnosed timely, treated properly and the patients are fully compliant, the long-term survival in patients with WD seems to be similar to the general population (20).

## Conclusion

WD can present in children as diffuse leukoencephalopathy mimicking Alexander disease. A 13-year-old boy with progressive dysarthria, bradykinesia, cognitive decline, and focal motor seizures showed symmetrical T2/FLAIR hyperintensities with cystic encephalomalacia in frontal white matter, restricted diffusion, and basal ganglia/brainstem involvement—a pattern strongly suggestive of a leukodystrophy. Whole-exome sequencing identified a homozygous ATP7B mutation (c.2333G > T, p.Arg778Leu), subsequently confirmed by low ceruloplasmin, elevated 24-hour urinary copper, and Kayser-Fleischer rings, which revised the diagnosis to Wilson's disease. This case highlights that Wilson's disease must be considered in the differential diagnosis of progressive bilateral frontal leukoencephalopathy in children, even when classic copper-related basal ganglia lesions are absent. Early copper screening and genetic testing are crucial to avoid diagnostic delay and enable timely chelation therapy. Clinical, laboratory, and neuroimaging integration is key to recognizing this treatable mimic of inherited leukodystrophies.

## Data Availability

The datasets presented in this article are not readily available because of ethical and privacy restrictions. Requests to access the datasets should be directed to the corresponding author.
